# Where is the gap after a 90 W/4 s very‐high‐power short‐duration ablation of atrial fibrillation?: Association with the left atrial‐pulmonary vein voltage and wall thickness

**DOI:** 10.1002/joa3.13009

**Published:** 2024-02-27

**Authors:** Moyuru Hirata, Koichi Nagashima, Ryuta Watanabe, Yuji Wakamatsu, Shu Hirata, Sayaka Kurokawa, Yasuo Okumura

**Affiliations:** ^1^ Division of Cardiology, Department of Medicine Nihon University School of Medicine Tokyo Japan

**Keywords:** atrial fibrillation, very high‐power short‐duration ablation, voltages, wall thickness

## Abstract

**Background:**

Although pulmonary vein isolation (PVI) for atrial fibrillation (AF) utilizing radiofrequency (RF) applications with a very high‐power and short‐duration (vHPSD) has shortened the procedure time, the determinants of pulmonary vein (PV) gaps in the first‐pass PVI and acute PV reconnections are unclear.

**Methods:**

An extensive encircling PVI was performed with the QDOT MICRO catheter with a vHPSD (90 W–4 s) in 30 patients with AF (19 men, 64 ± 10 years). The association of the PV gap sites (first‐pass PVI failure, acute PV reconnections [spontaneous reconnections or dormant conduction provoked by adenosine triphosphate] or both) with the left atrial (LA) wall thickness and LA bipolar voltage on the PVI line and ablation‐related parameters were assessed.

**Results:**

PV gaps were observed in 29 (6%) of 480 segments (16 segments per patient) in 17 patients (56%). The PV gaps were associated with the LA wall thickness, bipolar voltage, and the number of RF points (LA wall thickness, 2.5 ± 0.5 vs. 1.9 ± 0.4 mm, *p* < .001; bipolar voltage, 2.59 ± 1.62 vs. 1.34 ± 1.14 mV, *p* < .001; RF points, 6 ± 2 vs. 4 ± 2, *p* = .008) but were not with the other ablation‐related parameters. Receiver operating characteristic curves yielded that an LA wall thickness ≥2.3 mm and bipolar voltage ≥2.40 mV were determinants of PV gaps with an area under the curve of 0.82 and 0.73, respectively.

**Conclusions:**

The LA voltage and wall thickness on the PV‐encircling ablation line were highly associated with PV gaps using the 90 W/4 s‐vHPSD ablation.

## Graphical abstract

The left atrial (LA) voltage and wall thickness on the pulmonary vein (PV)‐encircling ablation line were highly associated with PV gaps when using the 90 W/4 s‐vHPSD ablation. The determined cut‐off values for predicting PV gaps were 2.40 mV for LA voltage and 2.3 mm for wall thickness, respectively. These findings provide valuable clinical insights into enhancing first‐pass PV isolation with the 90 W/4 s‐vHPSD ablation technique.

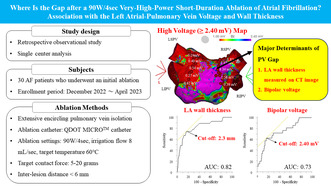

## 1 INTRODUCTION

Recently, there has been established evidence regarding the safety and efficacy of pulmonary vein isolation (PVI) of atrial fibrillation (AF) using high‐power short‐duration (HPSD) ablation techniques, typically involving a 40–50 W power and 10–15 s duration.^1^ A novel approach using very high‐power short‐duration (vHPSD) ablation with 90 W for 4 s using a QDOT MICRO™ catheter has emerged, aiming to further reduce the ablation time while maintaining the efficacy and safety.^2^ Clinical studies have shown that vHPSD ablation with this catheter can indeed shorten the total ablation duration, achieving a comparable effectiveness and safety to HPSD ablation.^3^, ^4^, ^5^ However, concerns have been raised regarding a slightly lower first‐pass PVI rate and higher acute pulmonary vein (PV) reconnection rate with the vHPSD ablation as compared to the HPSD ablation.^4^, ^5^ One hypothesis is that the increased rate of PV reconnection sites may be attributed to the thickened wall at the PV‐left atrial (LA) junction, as previous animal studies have demonstrated that vHPSD radiofrequency (RF) applications can create wider endocardial lesions but with a slightly shallower penetration as compared to HPSD ablation.^2^, ^6^ Additionally, our clinical research focused on exploring whether the presence of PV‐LA junction wall thickening or the high bipolar electrogram (EGM) voltage amplitude at the PV‐LA junction can explain most PV reconnection sites.^7^, ^8^ To investigate these hypotheses, our study aimed to examine the impact of the LA wall thickness and EGM voltage amplitude along the encircling PVI line on PV gaps following a vHPSD PVI ablation.

## 2 METHODS

### 2.1 Study design

We conducted a retrospective analysis of 30 consecutive patients (19 men, mean age 64 ± 10 years) with nonvalvular AF who underwent an initial RF catheter ablation procedure known as an extensive encircling pulmonary vein isolation (EEPVI) at the Nihon University Itabashi Hospital between December 2022 and April 2023. Prior to the ablation procedure, all study patients received adequate oral anticoagulation for a minimum of 1 month.

### 2.2 Computed tomography (CT)‐based measurement of the LA wall thickness

Multi‐detector helical CT with a 320‐row detector, dynamic volume CT scanner (Aquilion ONE; Toshiba Medical Systems, Tokyo, Japan) was performed 1–7 days before the ablation procedure. Scanning was performed at a slice thickness of 0.5 mm, gantry rotation time of 350 ms, tube voltage of 120 kV, and tube current of 300–580 mA for optimum detection of fine structures (resolution of approximately 0.3 mm). Landiolol was administered to maintain the patient's heart rate at <65 bpm, and nonionic iodinated contrast (Iomeron, Eisai Co, Tokyo, Japan) was injected at 0.07 mL/kg/s for 9 s. The timing of the image acquisition was determined by bolus tracking software; imaging was initiated when the contrast reached the LA. During the end‐expiratory phase, the volume acquisition was gated to 65%–75% of the R–R interval on the lead II electrocardiogram during sinus rhythm (SR) or an AF rhythm. Two patients were excluded from the study as those patients had an allergy to contrast media.

The wall thickness of the LA on the PVI line was measured as previously described.^7^, ^8^, ^9^ In brief, the acquired CT images were transferred to a workstation (ZIO M900 3.0; QUADRA; Amin Co., Ltd., Tokyo, Japan), where epicardial fat was detected and excluded from the wall of the PVI line by the assignment of Hounsfield units of −50 to −200. The wall thickness was measured at the following 16 segments: the anterior, posterior, and superior aspects of the right and left superior PVs (RSPV and LSPV, respectively), anterior and posterior carinas of the right and left PVs, and anterior, posterior and inferior aspects of the right and left inferior PVs (RIPV and LIPV, respectively). Coronal plane images were used for measurement at the superior and inferior aspects of the PVs, horizontal plane images for the anterior and posterior aspects of the PVs, and sagittal plane images for the anterior and posterior aspects of the PV carinas. At each location, 3 sites within 5 mm from the thickest part were measured with electronic calipers and averaged for the analysis. The mean LA wall thickness per patient was defined as the average LA wall thickness of the 16 PV segments per patient.

### 2.3 Electrophysiologic study and voltage mapping

An electrophysiologic study was performed with patients under conscious sedation achieved with dexmedetomidine, and fentanyl. After vascular access was obtained, a single transseptal puncture was performed, and intravenous heparin was administered to maintain an activated clotting time of >300 s. After two long sheaths (VIZIGO steerable sheath and SL0 sheath; St. Jude Medical Inc., St. Paul, MN) were inserted into the LA via a transseptal puncture, the 3‐dimensional (3D) geometry of the LA and four PVs was reconstructed by means of a CARTO3 mapping system (Biosense Webster, Diamond Bar, CA, USA). High‐density LA PVI line voltage mapping was performed during SR before the ablation. If the patient was in an AF rhythm, the voltage map was created after SR was restored by internal low‐energy electrical cardioversion (10–20 J). Six patients were excluded from the study because LA voltage maps could not be obtained because of the immediate recurrence of AF after cardioversion. Bipolar signals high‐pass filtered at 30 Hz and low‐pass filtered at 500 Hz were acquired by means of a multipole, multispline catheter with 2‐mm interelectrode spacing (OCTARAY NAV; Biosense Webster). As previously reported, at each of the 16 PV segments where the CT‐based measurements of the LA wall thickness on the PVI line were obtained, the voltages of the three sites closest to the ablation line were measured with electronic calipers and averaged for the analysis.^8^ The mean bipolar EGM voltage amplitude on the PVI line per patient was defined as the average voltage of 16 PV segments per patient.

### 2.4 Ablation procedure

The EEPVI was performed with a second‐generation irrigated catheter (QDOT MICRO™ catheter, Biosense Webster, Diamond Bar, CA) during SR (*n* = 24) or AF (*n* = 6) with a target contact force (CF) of 5–20 g, and an interlesion distance (ILD) <6 mm. A temperature‐controlled ablation was performed with the QDOT MICRO™ catheter and an RF generator (nGEN RF Generator, Biosense Webster) with an initial output power of 90 W for 4 s and an irrigation flow of 8 mL/min (Qmode+). The real‐time catheter‐surface temperature assessed by the six thermocouples situated at the surface of the ablating electrode tip was sent to the generator every 33 ms during the RF application and visualized in the bull's eye monitor. If the real‐time surface temperature increased to over the target temperature of 60°C, the power output of the RF automatically decreased until the temperature reached the target temperature. Completion of the EEPVI was confirmed by bidirectional (entrance and exit) block. PV entrance block was defined as elimination of the PV potentials, and PV exit block was defined as the absence of conduction to the LA during bipolar pacing from the OCTARAY catheter positioned at the PV ostia. At least 30 min after the EEPVI, 30 mg of adenosine triphosphate (ATP) was injected to detect any PV reconnections including spontaneous PV reconnections and PV dormant conduction.

If the first‐pass PVI failed or if an acute PV reconnection, defined as a spontaneous reconnection or dormant conduction provoked by a 30 mg ATP injection at least 30 min after the EEPVI, was seen, the segment with the gap in the PVI line was identified by the OCTARAY catheter, and categorized according to the 16 PV segments where the wall thickness was measured. A successful first‐pass PVI was defined as a complete PVI (absence of acute PV reconnections) achieved during the initial RF applications. The PV gap was defined as a first‐pass PVI failure, acute PV reconnection (spontaneous reconnection or dormant conduction), or both.

Additional RF energy was applied to the conduction gaps using the vHPSD (90 W/4 s) or HPSD settings (50 W) guided by an ablation index (AI) of 450–550, with irrigation flow rates of 4 or 15 mL/min (Qmode), until the ATP‐induced dormant conduction disappeared. The endpoint of the procedure was the completion of the EEPVI. Cavotricuspid isthmus ablation was performed when typical atrial flutter was induced by burst atrial pacing or observed clinically.

### 2.5 Follow‐up

Antiarrhythmic drugs were resumed after the ablation procedure at the operator's decision. All patients underwent routine follow‐up at our institution at 3 weeks and 3, 6 months after ablation or whenever they had any symptoms. Twelve‐lead electrograms were recorded at each visit, and 24‐h Holter recordings were obtained at 3 and 6 months after the ablation procedure. Recurrence was defined as any document of AF or atrial tachycardia (AT) of more than 30 s after the blanking period of 3 months.

### 2.6 Statistical analysis

Continuous variables are expressed as the mean ± SD values or median (25th percentile or 75th percentile), and categorical variables are expressed as the number and percentage of patients. Differences in the continuous variables were analyzed by the Student *t* test or Mann–Whitney *U* test, as appropriate. The chi‐square test was used to analyze the differences in the dichotomous variables unless the expected values in cells were <5, in which case a Fisher's exact test was used. A multivariate logistic regression analysis was performed to identify any factors affecting the PV gaps. Significant variables by univariate analysis including LA wall thickness, bipolar EGM voltage amplitude, and the number of the RF applications were entered into this model. All these statistical analyses were performed with JMP Pro 16 software (SAS Institute, Cary, NC). Receiver operating characteristic (ROC) curves were plotted to determine the cut‐off LA wall thickness and bipolar voltage on the PVI line for the prediction of PV gaps.

## 3 RESULTS

### 3.1 Patient characteristics

The clinical characteristics of the 30 study patients are summarized in Table 1. Twenty‐one patients (70%) had paroxysmal AF and 9 (30%) persistent AF. The median CHA_2_DS_2_‐VASc score was 2 (1, 4). The LA dimension and left ventricular ejection fraction were 38 ± 6 mm and 64 ± 8%, respectively. One patient had a common left PV ostium. All PVs were successfully isolated after the EEPVI in all patients. In total, 77 ± 13 ablation points were required for the EEPVI: 37 ± 8 points for the left PVs and 39 ± 8 points for the right PVs.

**TABLE 1:** Patient and ablation‐related characteristics.

	Total (*n* = 30)	PV gap (*n* = 17)	No PV gap (*n* = 13)	*p* value
Clinical characteristics				
Age (years)	64 ± 10	64 ± 12	66 ± 9	.63
Male gender	19 (63)	10 (59)	9 (41)	.70
BMI (kg/m^2^)	25 ± 5	26 ± 6	24 ± 4	.19
Paroxysmal AF	21 (70)	12 (71)	9 (70)	1.00
AF duration (months)	6 (5, 13.5)	12 (6.5, 25)	20 ± 21	.16
Hypertension	17 (56)	8 (47)	9 (69)	.28
Hyperlipidemia	7 (23)	3 (18)	4 (31)	.66
Diabetes mellitus	6 (20)	4 (23)	2 (15)	.67
Heart failure	7 (23)	4 (23)	3 (23)	1.00
History of stroke	3 (10)	2 (12)	1 (8)	1.00
Vascular disease	1 (3)	0 (0)	1 (8)	.43
CHA_2_DS_2_‐VASc score	2 (1, 4)	2 (1.5, 3.5)	2 (1.5, 4)	.88
Antiarrhythmic drug use	14 (46)	8 (47)	6 (46)	1.00
Class I	8 (26)	5 (29)	3 (23)	1.00
Class III	1 (10)	0 (0)	1 (7.6)	.43
Class IV	10 (33)	6 (35)	4 (30)	1.00
Echocardiographic measurements				
LVEF (%)	64 ± 8	66 ± 8	63 ± 9	.36
LAD (mm)	38 ± 6	39 ± 6	38 ± 5	.57
Treatment‐related variables				
Number of voltage mapping points	7342 ± 2932	7520 ± 2582	7192 ± 3296	.79
Bipolar voltage on the PVI line (mV)	1.40 ± 0.49	1.54 ± 1.21	1.19 ± 1.16	.006
LA wall thickness on the PVI line (mm)	1.9 ± 0.4	2.0 ± 0.4	1.9 ± 0.4	.31
Number of RF application points	77 ± 13	78 ± 15	74 ± 10	.47
LPV	37 ± 8	38 ± 9	36 ± 5	.44
RPV	39 ± 8	39 ± 2	38 ± 3	.69
PVI procedure time (min)	31.3 ± 16.9	39.4 ± 17.6	20.7 ± 7.8	.001

*Note*: Values are shown as the mean ± SD, median (25th, 75th interquartile range) or *n* (%).

Abbreviations: AF, atrial fibrillation; BMI, body mass index; LAD, left atrial dimension; LVEF, left ventricular ejection fraction; LPV, left pulmonary vein; PV, pulmonary vein; PVI, pulmonary vein isolation; RF, radiofrequency; RPV, right pulmonary vein.

### 3.2 Patient characteristics with and without PV gaps

**FIGURE 1:**
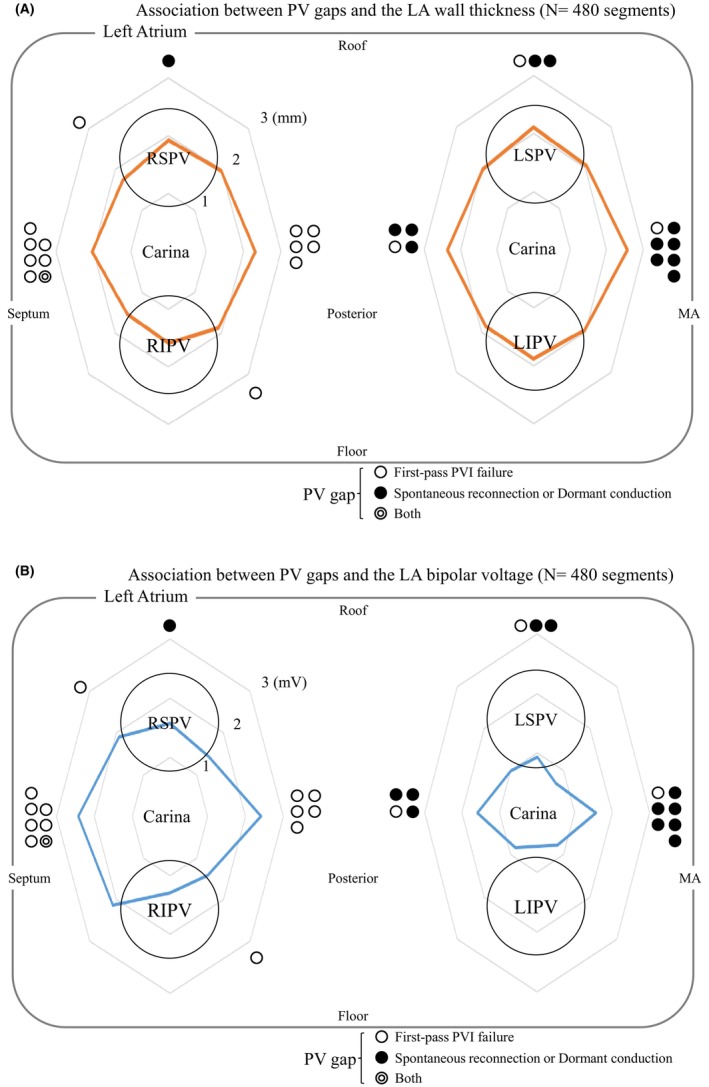
(A, B) Distribution of the PV gaps across the 16 PV–left atrium junction segments around the four PVs within the ablation line. The white circles indicate first‐pass PVI failure, and the black circles indicate spontaneous reconnections or dormant conduction provoked by adenosine triphosphate. Both a first‐pass PVI failure and spontaneous reconnection or dormant conduction (indicated by the double circle) were observed in 1 patient. LA, left atrium; LIPV, left inferior pulmonary vein; LSPV, left superior pulmonary vein; MA, mitral anulus; PV, pulmonary vein; PVI, pulmonary vein isolation; RIPV, right inferior pulmonary vein; RSPV, right superior pulmonary vein.

The mean number of voltage mapping points was 7342 ± 2932 points per patient. The first‐pass PVI was successful in 17 patients (56%) and 45 ipsilateral PVs (75%). The distribution of the PV gap sites across the 16 PV segments, LA wall thickness, bipolar EGM voltage amplitude on the ablation line, and ablation parameters are shown in Table 2 and Figure 1A,B. PV gaps were observed after the PVI in 27 of the 60 ipsilateral PVs (45%) and in 29 (6%) of the 480 PV segments; 13 patients (44%) and 17 segments (4%) had first‐pass PVI failures, and 9 (30%) patients and 13 segments (3%) had spontaneous reconnections or dormant conduction. The mean LA bipolar EGM voltage was higher (1.54 ± 1.21 vs. 1.19 ± 1.16 mV, *p* = .006) and there was a longer PVI procedure time (39.4 ± 17.6 vs. 20.7 ± 7.8 min, *p* = .001) in the patients with PV gaps (Table 1). However, the incidence of PV gaps was not associated with any of the other clinical parameters.

**TABLE 2:** LA wall thickness, bipolar voltage, FTI, total RF energy, and automatic titration of RF‐power by less than 60 W in the PV gap of the 16 PV‐LA segments.

	PV gap				LA wall thickness (mm)	LA bipolar voltage (mV)	FTI (gs)	Total RF energy (J)	Titratio*n* < 60 W (%)
	Total (*n* = 29)	First‐pass PVI failure (*n* = 17)	Spontaneous (*n* = 10)	DC (*n* = 3)					
LSPV									
Roof	3	1	2	0	1.9 ± 0.4	0.93 ± 1.31	66 ± 22	317 ± 22	3 (10)
Anterior	0	0	0	0	2.0 ± 0.4	0.70 ± 0.71	53 ± 17	318 ± 22	3 (10)
Posterior	0	0	0	0	1.7 ± 0.3	0.99 ± 0.89	81 ± 23	324 ± 17	3 (10)
LPV carina									
Anterior	7	1	4	2	2.3 ± 0.4	1.56 ± 1.34	56 ± 14	321 ± 14	3 (10)
Posterior	4	1	3	0	2.0 ± 0.5	1.59 ± 1.37	58 ± 17	314 ± 36	2 (7)
LIPV									
Anterior	0	0	0	0	1.9 ± 0.3	0.76 ± 0.58	56 ± 15	309 ± 61	4 (13)
Posterior	0	0	0	0	1.5 ± 0.3	0.82 ± 0.84	65 ± 24	318 ± 18	2 (7)
Floor	0	0	0	0	1.6 ± 0.4	0.56 ± 0.37	63 ± 20	307 ± 43	0
RSPV									
Roof	1	0	1	0	2.1 ± 0.3	1.58 ± 0.96	63 ± 20	317 ± 28	4 (17)
Anterior	1	1	0	0	2.0 ± 0.3	1.44 ± 1.15	57 ± 12	322 ± 17	3 (10)
Posterior	0	0	0	0	2.0 ± 0.4	1.90 ± 1.39	71 ± 21	321 ± 23	3 (10)
RPV carina									
Anterior	7	7	0	1	2.6 ± 0.5	2.41 ± 1.19	69 ± 18	319 ± 23	3 (10)
Posterior	5	5	0	0	2.4 ± 0.5	2.42 ± 1.39	61 ± 16	314 ± 36	5 (17)
RIPV									
Anterior	0	0	0	0	2.0 ± 0.3	1.41 ± 1.00	66 ± 19	317 ± 28	7 (23)
Posterior	1	1	0	0	1.9 ± 0.4	2.17 ± 1.24	65 ± 17	326 ± 14	1 (3)
Floor	0	0	0	0	1.9 ± 0.5	1.29 ± 0.79	66 ± 15	319 ± 23	1 (3)

*Note*: Values are shown as the mean ± SD, or *n* (%).

Abbreviations: DC, dormant conduction; FTI, force‐time integral; LA, left atrium; LPV, left pulmonary vein; LSPV, left superior pulmonary vein; LIPV, left inferior pulmonary vein; PV, pulmonary vein; PVI, pulmonary vein isolation; RF, radiofrequency; RPV, right pulmonary vein; RSPV, right superior pulmonary vein; RIPV, right inferior pulmonary vein.

### 3.3 Ablation‐related parameters at sites with and without PV gaps

**FIGURE 2:**
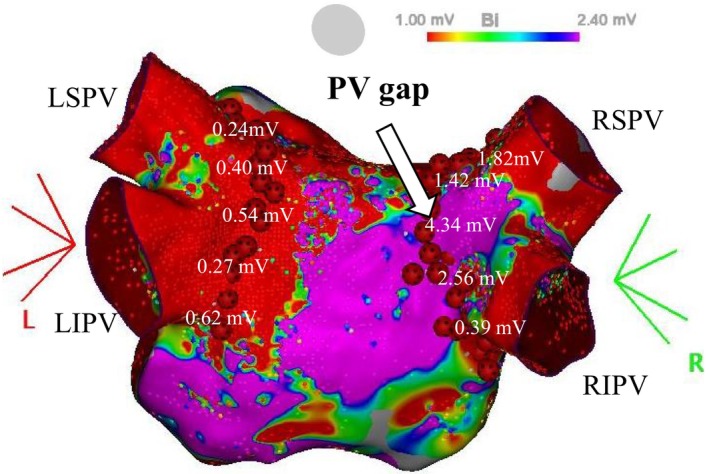
Representative PV‐left atrium bipolar voltage map. On the voltage map, the bright purple areas are defined as high‐voltage areas (≥2.40 mV), and the blue, green, yellow, orange, and red areas are those of a lower voltage (<2.40 mV). Ablation tags turn pink if the total RF energy is less than 329 J. In this case, a PV gap is observed in the posterior aspect of the RPV carina (white arrow). LIPV, left inferior pulmonary vein; LSPV, left superior pulmonary vein; PV, pulmonary vein; RIPV, right inferior pulmonary vein; RSPV, right superior pulmonary vein.

**FIGURE 3:**
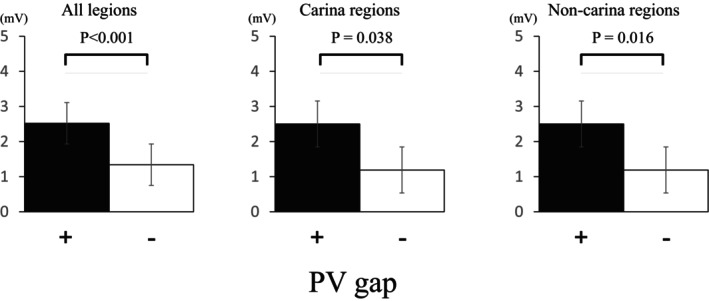
The PV–left atrium voltages in all regions, the carina region and noncarina region with and without PV gaps. The voltage is higher at sites with PV gaps than at sites with no gaps (all: 2.59 ± 1.62 vs. 1.34 ± 1.14 mV, *p* < 0.001; carina: 2.62 ± 1.38 vs. 1.85 ± 1.34 mV, *p* = 0.038; noncarina: 2.50 ± 2.72 vs. 1.19 ± 1.03 mV, *p* = 0.016). PV, pulmonary vein.

**FIGURE 4:**
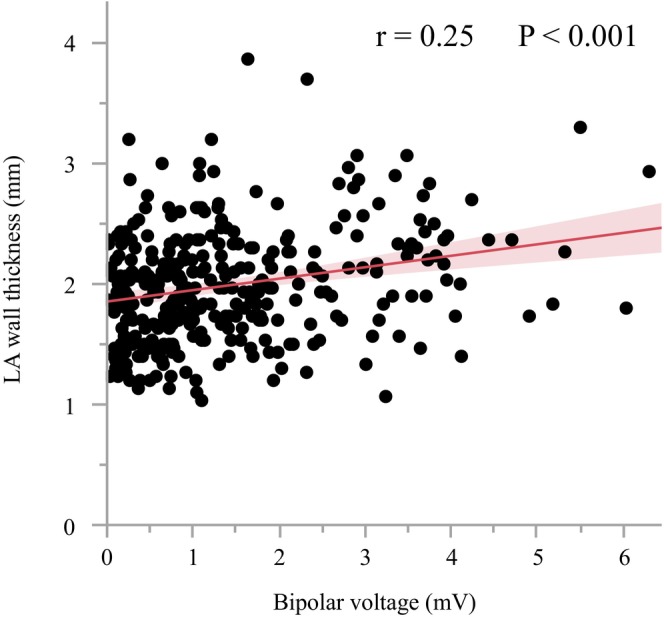
A scatter plot and regression line showing the relationship between the left atrial bipolar voltage and left atrial wall thickness on the PVI line.

PV gaps were more often observed in the carina regions than in the other segments (23 [19.1%] of the 120 PV carina segments vs. 6 [1.6%] of the 360 other segments, *p* < .001). Among those PV gaps, the first‐pass PVI failures were often observed at the right PV carina regions, and spontaneous reconnections and dormant conduction were often observed at the left PV carina regions. A representative LA voltage map that indicates the site of the PV gap is shown in Figure 2. The LA wall was thicker and bipolar EGM amplitude was higher at the sites with PV gaps (LA wall thickness, 2.5 ± 0.5 vs. 1.9 ± 0.4 mm, *p* < .001, bipolar EGM voltage amplitude, 2.59 ± 1.62 vs. 1.34 ± 1.14 mV, *p* < .001, Table 3). Similar results were observed when the voltage analysis was performed in the carina region versus the noncarina region (Figure 3). A modest correlation was found between the LA wall thickness and bipolar EGM voltage amplitude (*r* = .25, *p* < .001, Figure 4). The number of the RF applications was higher (6 ± 2 vs. 4 ± 2, *p* = .008) at the sites with PV gaps, but there were no significant differences in the maximum RF power, mean CF, mean impedance drops, force‐time integral, max temperature, total RF energy, and automatic titration of the RF‐power by less than 60 W between the sites with and without PV gaps. The multivariate analysis revealed that the LA wall thickness and bipolar EGM voltage amplitude on the PVI line were independent predictors of PV gaps (LA wall thickness, Odds ratio 6.04, 95% CI 2.23–16.3, *p* = .004; bipolar voltage, Odds ratio 1.57, 95% CI 1.12–2.22, *p* = .005), but the number of RF application points was not associated with PV gaps (Odds ratio 0.99, 95% CI 0.83–1.18, *p* = .91).

**TABLE 3:** Ablation‐related parameters at sites with and without PV gaps.

*n* = 480	PV gap (*n* = 29)	No PV gap (*n* = 451)	*p* value
LA wall thickness on the PVI line (mm)	2.5 ± 0.5	1.9 ± 0.4	<.001
Bipolar voltage on the PVI line (mV)	2.59 ± 1.62	1.34 ± 1.14	<.001
Number of RF application points	6 ± 2	4 ± 2	.008
Maximum RF power (W)	90.0 ± 0.1	89.8 ± 2.0	.49
Mean contact force (g)	16.1 ± 3.9	16.4 ± 5.1	.76
Mean impedance drop (Ω)	9.7 ± 1.9	9.9 ± 2.6	.72
FTI (gs)	62 ± 13	64 ± 19	.51
Max temperature (°C)	51 ± 3	51 ± 2	.24
Total RF energy (J)	316 ± 34	318 ± 28	.72
Automatic titration of RF‐power by less than 60 W	2 (6)	47 (11)	.54

*Note*: Values are shown as the mean ± SD, or *n* (%).

Abbreviations: FTI, force‐time integral; LA, left atrium; PV, pulmonary vein; PVI, pulmonary vein isolation; RF, radiofrequency.

### 3.4 Prognostic performance of the voltage and ablation‐related parameters for PV gaps

**FIGURE 5:**
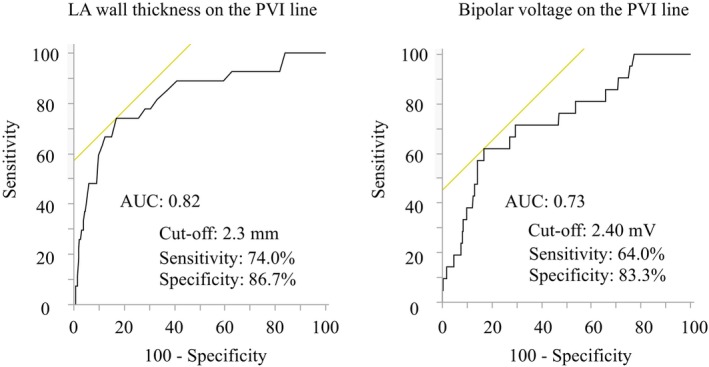
Receiver‐operating characteristic curves drawn for the prediction of PV gaps. The left atrial wall thickness and bipolar voltage on the encircling pulmonary vein isolation line provide a good predictive value, with AUC values of 0.82 and 0.73, respectively. AUC, area under curve; LA, left atrium; PVI, pulmonary vein isolation.

The prognostic ROC curves of the LA wall thickness and bipolar EGM voltage amplitude on the PVI line are shown in Figure 5. The LA wall thickness and the bipolar EGM voltage amplitude on the PVI line yielded the two strongest prognostic performances, with areas under the curves of 0.82 and 0.73, respectively. The best cut‐off values for predicting PV gaps were an LA wall thickness on the PVI line of ≥2.3 mm and a bipolar EGM voltage amplitude of ≥2.40 mV, respectively.

### 3.5 Ablation outcomes on the chronic phase

After the ablation procedure, 41 patients were maintained with antiarrhythmic drugs: bepridil in four, amiodarone in two, and a class I drug in one patient. After a median follow‐up of 9.8 (8.5, 10.7) months, two patients (6.6%) had AF/AT recurrences: Both of the two patients were AF recurrences. AF recurrence was not related to the presence of PV gap (two patients [11.7%] with PV gaps vs. 0% of patients without PV gaps, *p* = .51).

## 4 DISCUSSION

### 4.1 Main findings

This study had three major findings. First, only 56% of the patients achieved a successful first‐pass PVI, but when considering the individual PVs, 76% of them were isolated on the first attempt. Second, a first‐pass PVI failure was often observed at the right PV carina regions, while spontaneous reconnection or dormant conduction sites were at the left PV carina regions. Third, a high LA EGM voltage amplitude, thick LA wall, and the number of RF applications on the PV‐encircling ablation lines were associated with PV gaps. The cut‐off value of the LA voltage and wall thickness to predict PV gaps were determined to be 2.40 mV and 2.3 mm, respectively.

Our study demonstrated a significant correlation between the bipolar voltage and wall thickness in the pulmonary vein‐encircling ablation lines and the occurrence of PV gaps. Over the past two decades, the durability of the PVI has become a crucial factor in achieving successful chronic outcomes in AF treatment. To minimize PV reconnections, technologies to fully “register” actual anatomic images and to monitor CF sensing have evolved, and more recently, the HPSD ablation strategy has been a promising approach to improve the durability and safety of the PVI while reducing the procedural time.^1^, ^10^ Despite these advancements, recent reports have indicated that acute PV reconnections still occur in 4%–38% of patients who have undergone a PVI.^1^, ^5^, ^10^ It is important to note that while advancements in technologies aim to standardize and generalize physicians' skills and experiences, there is limited information available regarding the underlying patient factors such as the tissue characteristics, including the wall thickness or diseased tissues. Recent reports have highlighted the significance of the LA wall thickness as a factor contributing to PV reconnections.^2^, ^7^, ^8^ The vHPSD ablation with the QDOT MICRO™ catheter was initially developed to allow for more efficient and shorter procedures, utilizing 90 W for 4 s.^4^ However, our study, similar to several recent studies,^4^, ^5^ revealed a lower first‐pass PVI rate of 56% in patients as compared to the standard HPSD ablation. When examining the first‐pass PVI failure rate among the PV segments, it was observed in only 29 (6%) out of the 480 PV segments. Several animal studies have reported that a 90 W/4 s‐vHPSD ablation creates wider lesions on the endocardial atrial tissue, but slightly shallower than that with the 50 W HPSD ablation.^2^, ^6^ The shallower lesions observed with the 90 W/4 s‐vHPSD ablation may help explain the lower first‐pass PVI rate. Our study's findings support this, as we observed a strong association between a thickened LA wall and PV gaps, but none of the other clinical parameters were associated with it.^8^ Additionally, the LA bipolar EGM voltages were also associated with PV gaps, likely because of their modest correlation with the LA wall thickness.^8^, ^11^ We found an important finding that the cut‐off values of the LA bipolar EGM voltages and wall thickness on the PVI line for predicting PV gaps were ≥2.40 mV and ≥2.3 mm, respectively. Previous studies conducted in vitro or in vivo have demonstrated a lesion depth of 3.1–3.6 mm with the 90 W/4 s‐vHPSD ablation.^2^, ^6^, ^12^ Although our wall thickness cut‐off of ≥2.3 mm was slightly shallower, it can be considered reasonable when taking into account the complex anatomical structures and the impact of respirations and cardiac movements during clinical ablation procedures. Additionally, our group conducted similar evaluations with a different multispline mapping catheter (Penta‐Ray NAV; Biosense Webster) for the cut‐off values of the voltages on the posterior wall lines created using an AI‐guided ablation with a 3.5‐mm open‐irrigated‐tip SmartTouch catheter (power: 45 W [25–35 W when the esophageal temperature rose], target CF > 10 g, target AI: 400–450, ILD < 6 mm).^13^ We also evaluated the voltages on the PVI line (power: 25–30 W, target CF > 10 g, FTI > 400 gs, ILD <6 mm).^8^, ^11^ Those evaluations identified cut‐off values of ≥2.64 mV for the voltages to predict failure sites on the posterior wall lines and a voltage cut‐off of ≥2.69 mV for PVI failure sites. Therefore, the different ablation settings resulted in slightly higher voltage cut‐off values, however, different mapping catheter validations would be needed,^14^ suggesting there would be similar or shallower lesions with the 90 W/4 s‐vHPSD ablation as compared to the AI‐guided 45 W‐HPSD ablation or FTI‐guided 25–30 W‐standard power ablation.^8^, ^11^, ^13^

### 4.2 Clinical implications

This study demonstrated no difference in the standard patient characteristics between the patients who did and did not have PV gaps, but notably, there was a significant discrimination ability of the wall thickness and bipolar EGM voltages for predicting PV gaps in each PV segment. Those findings suggest that it is important to predict postablation PV gaps on a per‐segment basis rather than per patient. Therefore, preacquired CT images of the LA wall thickness are necessary to predict the specific sites of postablation PV gaps. However, wall thickness measurements do not provide a real‐time assessment of these variables, and accurately measuring the wall thickness just underneath the ablation tags is limited because of technical difficulties in incorporating such information into the 3D mapping system. Specific software that detects the wall thickness and integrates it into the CARTO mapping system could be more feasible in this regard.^15^ On the other hand, the LA EGM voltage amplitude is a simple real‐time variable that can be obtained at sites accurately mapped in 3D. This study found that increasing the number of RF applications on the PVI line was not related to acute PV reconnection sites. Therefore, alternative ablation settings, such as HPSD ablation with an AI tailored by the bipolar voltage or wall thicknesses should be considered in areas with a bipolar EGM voltage amplitude of >2.40 mV. The combination of a vHPSD and HPSD ablation might improve the first‐pass PVI rate and shorten the PVI procedure time. More recent studies with vHPSD have reported a higher first PVI rate of 67%–83.9%, which is statistically similar to that with AI‐guided HPSD ablation.^16^, ^17^, ^18^ Most of these studies set an ILD of ≤4 mm (so called the “very‐close protocol”),^17^, ^18^, ^19^ while ours was <6 mm. Therefore, strictly setting an ILD of 3–4 mm for thick walls might be another option to improve first PVI rate. Further studies, especially prospective studies, are needed to identify the optimal strategies that make use of the LA bipolar EGM voltages in order to reduce the risk of PV gaps.

In terms of mid‐term outcomes in the chronic phase, the success rate reached 93.4% during a median follow‐up of 9.8 (8.5, 10.7) months. The occurrence of AF/AT recurrences was not found to be associated with acute PV gaps. This suggests that, even if PV gaps occur acutely, the application of additional touch‐up lesions to these gaps could minimize AF/AT recurrences. The FAST AND FURIOUS PVI study, as mentioned earlier,^17^ also highlighted a superior 12‐month success rate in vHPSD ablation using the very‐close protocol compared to conventional AI‐guided ablation (78% vs. 64%, *p* = .142). Notably, in repeated assessments of recurrent AF cases, vHPSD using the very‐close protocol demonstrated significantly better PV durability than conventional AI‐guided ablation.^17^, ^19^ Taken together, it is suggested that vHPSD may achieve sufficient chronic PV durability and success rates, especially when efficiently utilizing techniques such as narrower ILD or applying additional touch‐up lesions to PV gaps.

### 4.3 Limitations

First, this study was conducted retrospectively in a patient group that was not large. Patient characteristics that might have influenced the study results were not controlled for. Nonetheless, comparisons were made between the ablation sites, and there was a large enough number of those sites to observe the lesion efficacy. A study in a larger group of patients could alter the LA voltage cut‐off values we found that predicted acute PV reconnections. Second, this study primarily focused on the acute success rate of only the vHPSD ablation; thus, it remains unclear whether PV gaps and long‐term AF/AT recurrence rate with vHPSD will be higher than those with conventional HPSD ablation. An randamozed control trial comparing vHPSD and HPSD ablation in the long‐term follow‐up is warranted to clarify these points. Nevertheless, reducing the acute failure rate is clinically crucial as it can lead to a decrease in the ablation time in those without PV gaps, as observed in this study. Additionally, it has the potential to prevent insufficient lesions caused by edema resulting from repeated ablation applications to PV gaps. Third, PV gaps might be attributed to multiple factors such as operator factors, including the RF energy output, CF, and catheter stability, and the patient factors, including the voltage and wall thickness of the LA junction. The combination of these parameters should be practically considered to increase the lesion durability.

## 5 CONCLUSIONS

The LA voltage and wall thickness on the PV‐encircling ablation line were highly associated with PV gaps using the 90 W/4 s‐vHPSD ablation. Therefore, in areas where the LA voltage is high or the wall thickness is thickened based on preacquired CT images, alternative ablation settings, such as an AI‐guided HPSD ablation tailored to the LA voltages and/or wall thickness, should be considered to create more durable lesions.

## CONFLICT OF INTEREST STATEMENT

Authors declare no conflict of interests for this article.

## ETHICS STATEMENT

The studies involving human participants were performed according to protocols approved by the Institutional Review Board of Nihon University Itabashi Hospital (RK‐230314‐14).

## DISCLOSURES

Dr. Okumura has received research funding from Biosense Webster, Inc., scholarship donation from Boston Scientific Japan, and Endowed Courses from Boston Scientific Japan, Japan Lifeline, Fukuda Denshi, Abbott Japan, BIOTRONIK Japan, Medtronic Japan. K.N. has received research funding and accepted remuneration from Johnson & Johnson K.K. Other authors: No disclosures.

## Data Availability

The data that support the findings of this study are available on request from the corresponding author. The data are not publicly available because of privacy or ethical restrictions.
